# Supplementary material for: Togaram Ensures Axial Alignment of the Sperm Neck
Source: bioRxiv. 2026 Apr 17:2026.04.15.718719. Preprint. [Version 1] doi: 10.64898/2026.04.15.718719 (PMC13104979; doi:10.64898/2026.04.15.718719)
Supplement: 1 [file NIHPP2026.04.15.718719v1-supplement-1.pdf]

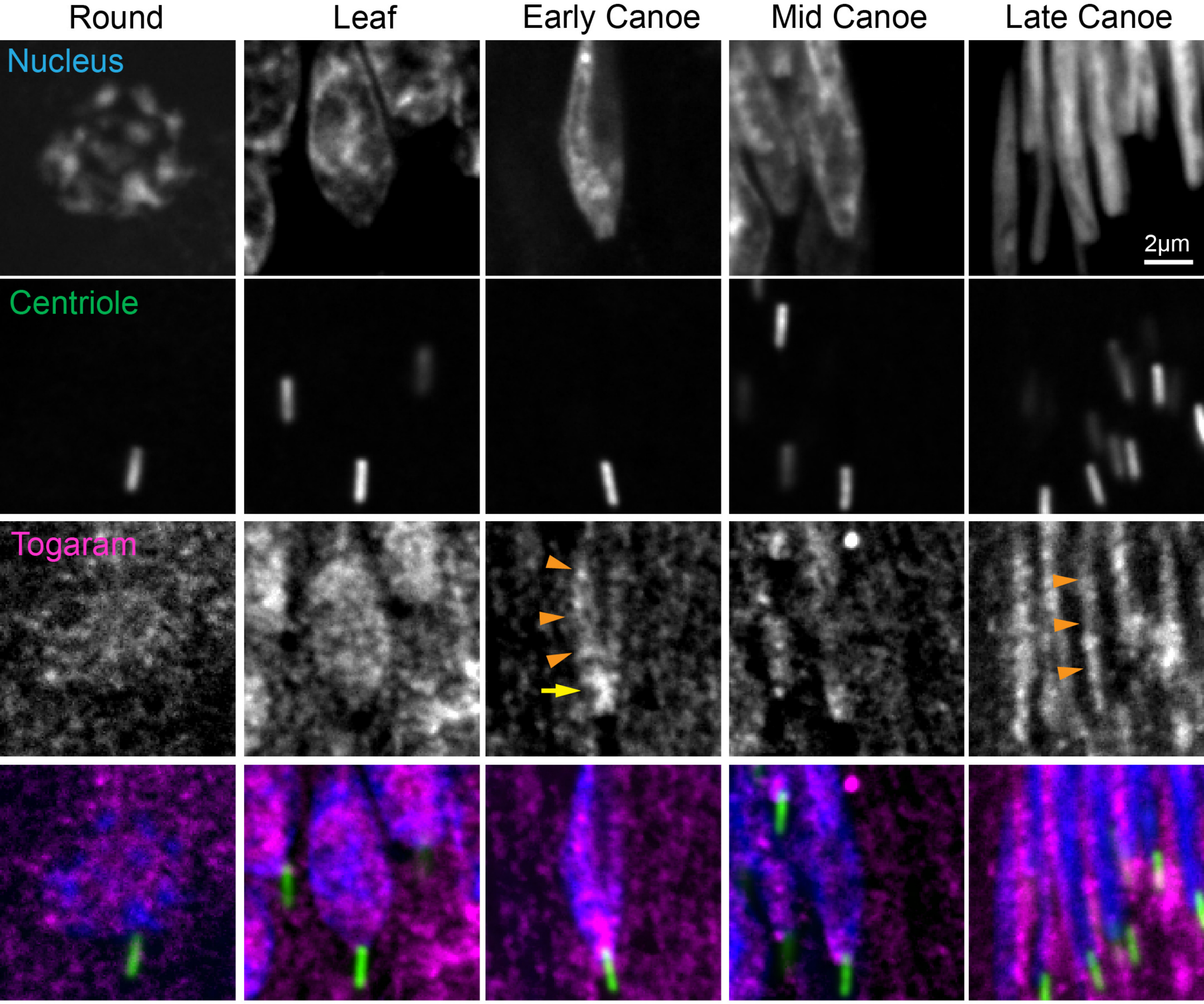

Figure S1, Burns et al.

bioRxiv preprint doi: https://doi.org/10.64898/2026.04.15.718719; this version posted April 17, 2026. The copyright holder for this preprint (which was not certified by peer review) is the author/funder. This article is a US Government work. It is not subject to copyright under 17 USC 105 and is also made available for use under a CC0 license.

Figure S1: Togaram dynamically localizes to sperm nucleus and neck

Representative images showing wild-type spermatids during indicated developmental stages. Spermatids are labeled for the nucleus (DAPI, blue), centriole (PACT::GFP, green), and Togaram (Toga, magenta). Yellow arrow denotes neck localization. Orange arrowheads denote nuclear localization. Scale bar: 2 μm.

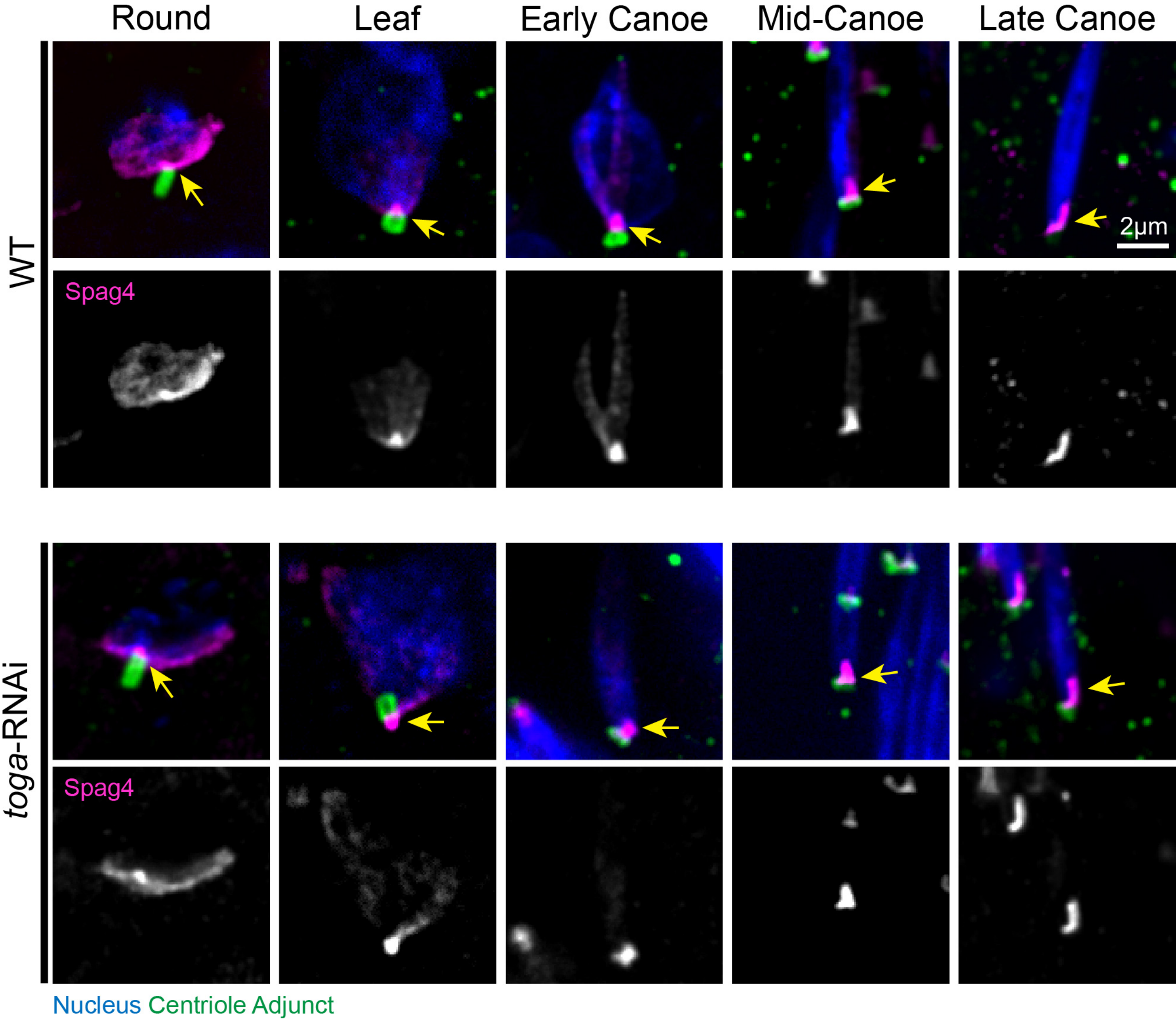

Figure S2, Burns et al.

bioRxiv preprint doi: https://doi.org/10.64898/2026.04.15.718719; this version posted April 17, 2026. The copyright holder for this preprint (which was not certified by peer review) is the author/funder. This article is a US Government work. It is not subject to copyright under 17 USC 105 and is also made available for use under a CC0 license.

Figure S2: Togaram does not disrupt Spag 4

Representative images showing wild-type (top) and toga-RNAi (bottom) spermatids during indicated developmental stages. Spermatids are labeled for the nucleus (DAPI, blue), the centriole adjunct (Asl, green), and Spag4 (magenta). Yellow arrows indicate the HTCA. Scale bar: 2 μm.

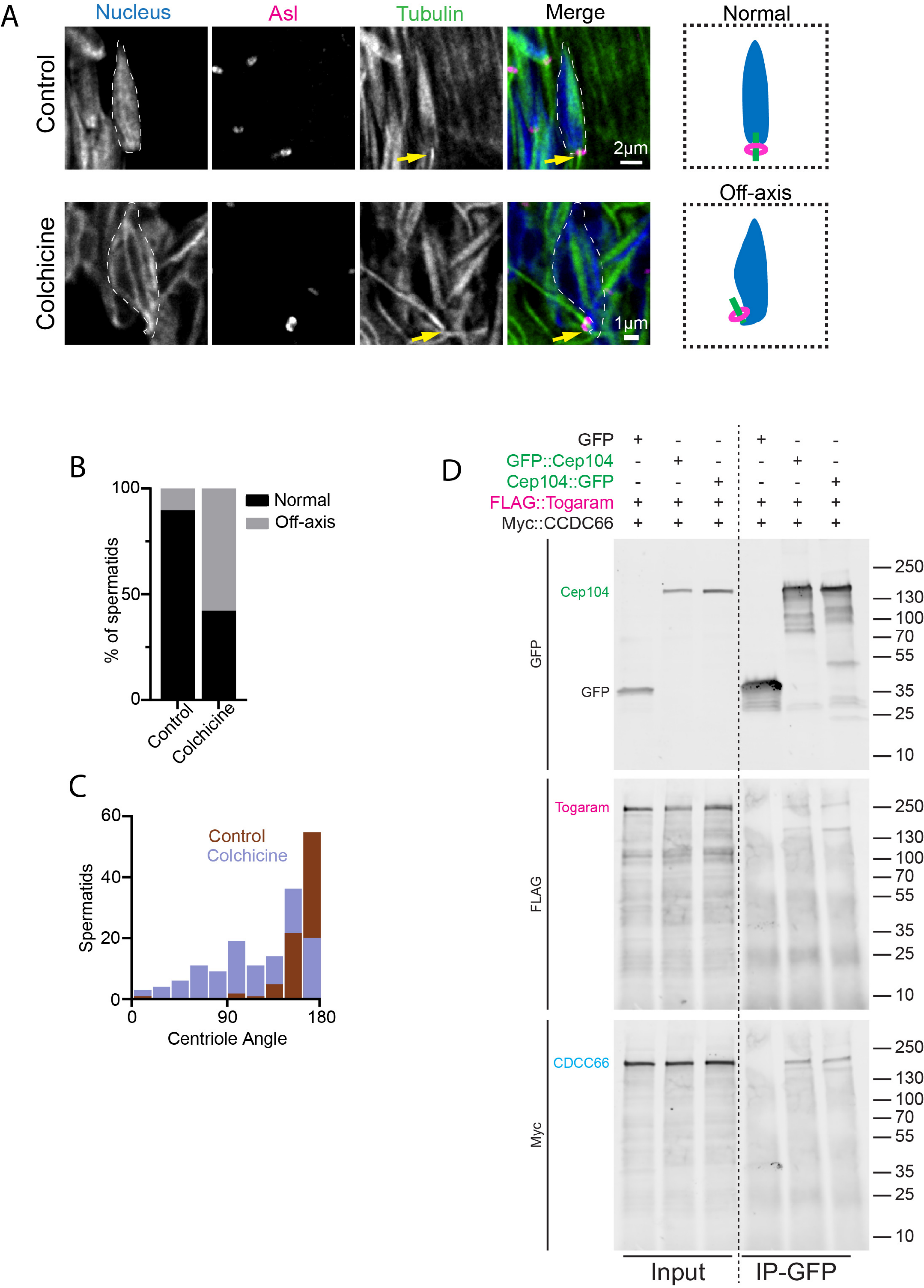

Figure S3, Burns et al.

bioRxiv preprint doi: https://doi.org/10.64898/2026.04.15.718719; this version posted April 17, 2026. The copyright holder for this preprint (which was not certified by peer review) is the author/funder. This article is a US Government work. It is not subject to copyright under 17 USC 105 and is also made available for use under a CC0 license.

Figure S3: Stable spermatid microtubules are required for sperm neck alignment

(A) Representative images showing control (top) or colchicine treated (bottom) Canoe stage spermatids. Spermatids are labeled for the nucleus (DAPI, blue), CA (Asl, magenta), and tubulin (ubi-GFP::tubulin, green). Yellow arrow indicates neck region. Cartoons depict spermatids with centrioles that are Normal or Off-axis in relation to nucleus. Scale bar: 2 µm (top), 1µm (bottom). (B) Quantification of control (n=86) and colchicine treated (n=133) spermatids with various alignment phenotypes. (C) Quantification of attached centriole angle to nucleus in control (n=86) and colchicine treated (n=133). (D) Raw gel images corresponding to Figure 5B. Both N- and C-terminally GFP-tagged Cep104 (green) binds Togaram (pink) and CCDC66 (blue). S2 cells were co-transfected with the indicated plasmids and anti-GFP co-IPs were prepared from cell lysates. Western blots of inputs and co-IPs were probed for GFP, Flag, and Myc.

bioRxiv preprint doi: https://doi.org/10.64898/2026.04.15.718719; this version posted April 17, 2026. The copyright holder for this preprint (which was not certified by peer review) is the author/funder. This article is a US Government work. It is not subject to copyright under 17 USC 105 and is also made available for use under a CC0 license.

Table S1. Qualitative GFP localization screen

Stock #	Gene Name	Localization
59974	CG10462	No significant signal
59975	CG10631	No significant signal
68659	CG10979	No significant signal
56785	CG12155	No significant signal
66590	CG1233	Head of mature sperm
92384	CG14441	Head of mature sperm
60235	CG1632	Head of mature sperm
6843	CG1640	Mitochondria
50877	CG1677	Head of mature sperm
63153	CG17349	Tails of mature sperm
67660	CG2199	Nuclei of spermatogonia
81263	CG2712	Head of mature sperm
60175	CG31183	No significant signal
60148	CG3339	Distal end of round stage spermatids centrioles
61775	CG34357	Head of mature sperm
50826	CG3939	No significant signal
65336	CG42399	Sperm neck region
92370	CG4496	No significant signal
50855	CG8036	No significant signal
50834	CG8209	Cytoplasm of spermatocytes
50802	Cindr	Muscle tissue and individualization cones
60190	Clip-190	Nuclear envelope of round stage spermatids and tails
58447	Don juan	Tails of sperm
60193	Karst	No significant signal
58406	Protamine B	Head of elongating and mature sperm
39647	Rhea	Head of mature sperm
91747	Spc105R	No significant signal
59827	Spire	No significant signal
60258	Sprint	No significant signal
60525	Obscurin	No significant signal
